# Nanoparticle-on-mirror pairs: building blocks for remote spectroscopies

**DOI:** 10.1515/nanoph-2022-0521

**Published:** 2022-10-27

**Authors:** Huatian Hu, Yuhao Xu, Zhiwei Hu, Bowen Kang, Zhenglong Zhang, Jiawei Sun, Yang Li, Hongxing Xu

**Affiliations:** School of Electronics and Information Engineering, Shenzhen University, Shenzhen 518060, China; Hubei Key Laboratory of Optical Information and Pattern Recognition, Wuhan Institute of Technology, Wuhan 430205, China; School of Physics and Technology, Wuhan University, Wuhan 430072, China; Wuhan Institute of Quantum Technology, Wuhan 430206, China; School of Physics and Information Technology, Shaanxi Normal University, Xi’An, China

**Keywords:** nanoantenna, nanoparticle on mirror, remote spectroscopy, surface plasmon polaritons

## Abstract

Surface-enhanced spectroscopies, such as surface-enhanced Raman scattering (SERS), fluorescence (SEF), circular dichroism, etc., are powerful tools for investigating nano-entities with high sensitivities. Owing to the giant local electric field confined in a plasmonic nanogap, nanogap-enhanced spectroscopies could detect samples with ultralow concentrations, even down to the single-molecule level for SERS and SEF. This great ability to detect analytes with ultralow concentrations provides opportunities for early diagnosis and monitoring in modern biomedicine. However, local laser excitations would inevitably bring about unwanted disruptive background perturbations, local heating, and the consequent geometry reshaping and biological analyte damages. Remote spectroscopies avoiding direct laser exposure to the samples can be treated as remarkable solutions. Here, we combined the nanoparticle-on-mirror (NPoM) family with the philosophy of remote spectroscopy to construct so-called “NPoM pairs” structures. They consist of two identical NPoMs with matched resonances yet separate functions either as receiving or transmitting antennas. A figure of merit for evaluating the remote spectroscopies was put forward, which accounts for the efficiencies in three processes, i.e., receiving, transporting, and transmitting. In addition, we experimentally demonstrated the performances of these NPoM pairs by proof-of-principle applications on the remote SERS and SEF. The optical access of the spectral information in these NPoM pairs both locally and remotely manifests themselves as fundamental building blocks for remote spectroscopies.

## 1 Introduction

Plasmonic nanogaps can significantly boost the light–matter interaction and amplify the plasmon-driven processes by concentrating light into a nanoscale hotspot (volume even down to 1 nm^3^ scale) with intense local field enhancement [1]. The plasmonically-powered spectroscopies, such as surface-enhanced Raman scattering (SERS), fluorescence (SEF), and circular dichroism, could push the conventional spectroscopies into the nanoscale with ultrahigh sensitivity. Remarkable single-molecule level SERS [2, 3] and SEF [4–6] have been widely achieved, providing great opportunities for early diagnosis and monitoring in modern biomedicine. Those spectroscopies are optically-driven, where the localized plasmons enhance the light signal which carries the information imprinted by the vibrational state, energy band, etc. These surface-enhanced spectroscopies reflecting the inherence within the matter serve as fundamental tools for exploring the physical and biochemical world.

Plasmonic nanoparticle-on-mirror (NPoM) is a family of nanostructures that includes several subsets such as nanosphere-on-mirror (NSoM) [7], nanocube-on-mirror (NCoM) [8], and nanowire-on-mirror (NWoM) [9], etc. They are shining structures for plasmon-enhanced spectroscopies and literally consist of metallic nanoparticles deposited on the metal film spaced by ultrathin dielectric nanogaps. With precisely controllable gap thickness, the easily fabricable NPoM acts like subwavelength nanocavities with highly intensive and low-loss localized plasmons [1, 8]. Giant Purcell factors, quantum yield, together with outstanding far-field emitting directions were observed [10, 11]. Thus, these versatile platforms have been widely used for amplifying the light–matter interaction [11–15] and accessing optical spectroscopies [10, 16], [17], [18].

Shining a laser on the nanostructures and collecting the *in-situ* responding signals is the universal route for locally accessing the spectra and investigating the light–matter interaction therein. However, direct excitation could also cause redundant heat effects and structural reshaping [18–22]. Besides, the background noise accompanied is also what we seek to exclude [23]. These drawbacks bring many disadvantages to accurate spectral sensing, especially for photonics diagnosis in biological systems. Remote spectroscopies consisting of well-separated receiving and transmitting nanoantennas are promising candidates for eliminating unwanted background noise and preventing the sample from direct laser exposure. Due to the excitation-collection-separated nature, remote configurations may boost the signal-to-noise ratio and prevent sample damage [23, 24]. The idea of remote spectroscopy could be traced back to various setups, including nanowire [25], nanowire-nanoparticle systems [24, 26, 27], nanowire bundles (arrays) [28], NWoM-NCoM matched pair [23], etc. The basic operating principle of these remote setups is to excite surface plasmons at receiving part, waveguide the propagating plasmons to the transmitting part, interact with the sample and deliver the spectral signal from that end. However, the in-coupling efficiency from the free space into the nanowire surface plasmon polaritons (SPPs) by end-scattering is relatively low (∼4%) [29]. Whereas the in-coupling efficiency from an NPoM nanoantenna to the SPPs on the film could be much higher (∼20%) due to the matched impedance between localized plasmons and SPPs [23, 30]. Note that the NPoMs could have high field enhancement and outstanding far-field radiation lobe [10], these film-coupled nanostructures, therefore, are superior configurations for implementing remote sensing.

In this work, we systematically analyze and compare different NPoM-pair schemes that harbor two distantly-separated identical NPoM nanoantennas. These identical pair structures are excellent in investigating energy transfer and entanglement due to the identical resonances and SPPs-powered inter-particle interaction [31]. After calculating the efficiencies (cross-sections) of generating SPPs, field enhancements, and remote Purcell factors in various nanoantennas, we experimentally choose NCoM pairs as a characteristic proof-of-principle demonstration. By combining NCoM pair and monolayer two-dimensional material-WS_2_, we experimentally demonstrate how the light waveguiding/transmitting, remote SERS, and remote SEF work in such a remote-detection system, step by step. Through massive measurements, the dependence of the remote sensing performance on the separation between nanoantennas was experimentally explored. By this means, we reveal that the NPoM pairs are solid building blocks for efficient remote nanogap-powered spectroscopies. Moreover, the compatibility of these nano units with two-dimensional materials further expands their potential applications in quantum information [32], biophotonics sensing [33], and *in vitro* diagnostics [34].

## 2 Results and discussion

**Figure 1:**
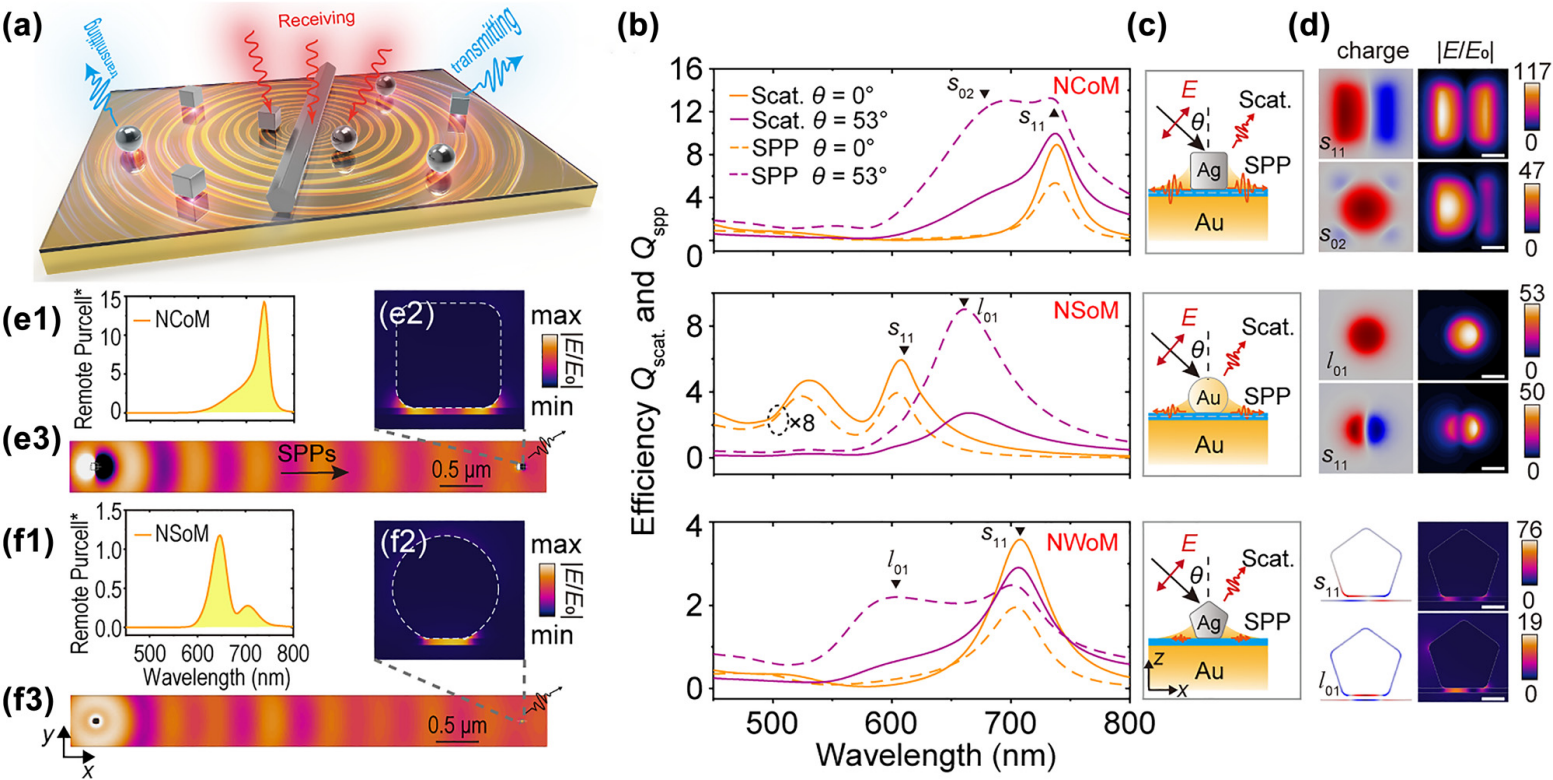
Optical properties of NPoM pairs family. (a) The schematic of NPoM pairs for remote spectroscopic purposes. Each kind of NPoM could be treated as receiving and transmitting antennas. (b) The efficiencies of scattering *Q*_scat_ and SPPs conversion *Q*_spp_ of various configurations: Upper panel (Ag NCoM), middle panel (Au NSoM), and lower panel (Ag NWoM). In (b), normal (yellow) and oblique (purple) incidences were checked and efficiencies of scattering (solid) and SPPs (dashed) were calculated. Schematics were shown in (c). (d) The charges and electric field enhancement of various resonances that occurred in (b). The charge and electric field of NPoM and NCoM were taken at the surface in the middle of the gap parallel to the film, while those of NWoM were from the *xz-*cross section. The white scalebars represent 25 nm. The remote sensing properties of NCoM (e1–e3) and NSoM (f1–f3) were indicated by their remote Purcell factors (*F*_P_^*^) and the remote field (e2, e3, f2, f3) excited by a dipole in receiving antennas.

NPoM families and their efficiencies. The schematic of the excitation-collection-separated NPoM pairs is shown in Figure 1a, where the three most-studied structures are shown and compared, i.e., NSoM, NCoM, and NWoM. As long as having matched resonances, every two nanoantennas could be considered as a functional pair, either as receiving or transmitting antenna. These receiving and transmitting nanoantennas were bridged by the SPPs sustained on the metal film. That is, as shown in Figure 1a, (1) the receiving nanoantenna captures light and transforms it into near-field SPPs; (2) the transmitting nanoantennas could be excited by these SPPs and transformed the near-field plasmons into far-field photons which carry the information of the spectra signals.

The sample to be measured could be placed to interact with plasmons either at receiving or transmitting parts: (1) if it is inserted at the nanogap of the receiving antenna, the launched SPPs from receiving antenna would carry the spectral signals (e.g., Raman, fluorescence, etc.) and the transmitting part would operate solely as amplification and broadcaster. This setup would prevent background noise due to stray illumination. (2) if the sample is placed at the transmitting antenna, which is most studied [24], the receiving part would solely act as an in-coupler, while the vertical electric field accompanying the SPPs would efficiently drive the transmitting nanoantenna to interact with the analytes and give birth to the far-field signals. This laser-sample-separated geometry could not only avoid background noise but also help deal with excessive heating and potential sample damage.

Without loss of generality, we chose the most-used ingredients: Ag nanocube, Au nanosphere, Ag nanowire, and Au film. They had comparable dimensions: Ag nanocube and Au nanosphere are 80 nm large, while the nanowire has a diameter of 80 nm and is treated as a pentagon in a 2D model. They were all placed on the ultrasmooth Au film with a 5 nm alumina spacer (refractive index *n* = 1.5) as a nanogap. To compare and estimate the coupling efficiency of those nanostructures, we calculate the efficiency of converting incident photons into the scattering (*Q*_scat_) and SPPs (*Q*_spp_). The efficiencies [35] are defined as *Q*_scat_ = *W*_scat_/(*I*_0_ × *S*_a_), and *Q*_spp_ = *W*_spp_/(*I*_0_ × *S*_a_), where the *I*_0_ is the incident intensity, and *S*_a_ is the particle’s geometric cross-sectional area. *W*_scat_ and *W*_spp_ are the net rates at which the electromagnetic energy (for scattering and SPPs, respectively) cross the surface *A*: *W*_scat_ = *∫* **S**_scat_ ⋅ **n** *dA*, *W*_spp_ = *∫* **S**_spp_ ⋅ **n** *dA*. **S**_scat_ and **S**_spp_ are their respective Poynting vectors, and the whole calculations were performed with a near-to-far field transformation [30] (details see methods). These efficiencies indicate how many times the cross-section of the scattering and SPPs conversions are larger than the geometric cross-section. The larger the efficiencies *Q*_scat_, *Q*_spp_ are, the more energy would be converted into scattering and SPPs, respectively.

Figure 1b compares the *Q*_scat_ and *Q*_spp_ of three comparable geometries (NCoM, NSoM, and NWoM) under normal (*θ* = 0°) and oblique (*θ* = 53°) excitations, shown in Figure 1c. We could find from the spectra that NCoM (Figure 1b, upper panel) has both larger scattering and SPPs efficiencies, i.e., *Q*_scat_, *Q*_spp_, than NSoM (middle panel) and NWoM (lower panel). Besides, oblique excitation (purple solid and dashed lines) and normal excitation (yellow solid and dashed lines) give birth to different plasmonic modes [8, 23, 36], [37], [38]. That is, (1) normal incident effectively excited the *s*_11_ cavity modes for all three structures (higher cavity mode was also shown for NSoM), due to the mode’s parity and symmetry. (2) apart from those cavity modes, the oblique incident could also excite antenna modes, in which the vertical bonding dipole-dipole modes (or *l*_01_ in NSoM) were most prominent. Higher cavity mode *s*_02_ was also observed in NCoM excited by the oblique incident [37]. Those resonances were identified by the charge distributions in Figure 1d.

Both scattering and SPPs efficiencies were highly enhanced by the resonances, disregarding the origin of the resonances: cavity-like modes and antenna-like modes all show great enhancements [38]. However, due to the morphology of the NSoM, its efficiencies under oblique excitations are much higher than the normal incidence (the normal-incident results were multiplied by 8 folds for clearer comparison in Figure 1b middle panel). We could roughly estimate the in-coupling efficiency *η* from the photon to the SPPs by comparing the SPPs conversion cross-section *Q*_spp_ × *S*_a_ to a waist area of the hypothetic incident beam (details see Methods). For the most efficient SPPs excitation (highest *Q*_spp_ for every configuration), the conversion efficiencies from photon to SPPs via three nanostructures are (details see Methods), 26% (NCoM), 14% (NSoM), and 30% (NWoM), respectively. Here we assumed the laser spot has a diameter of wavelength (estimated from diffraction limit), where the assumptive wavelength is 650 nm (near the highest *Q*_spp_ for all three structures for comparison, see purple dashed lines in Figure 1b). This result could be counter-intuitive, because one may believe the geometric overlap between laser spot and NWoM (12%) should be much larger than that with NCoM (1.9%) so that its in-coupling efficiency should have been larger. But this is not the case here because NCoM has a much larger efficiency *Q*_spp_ which compensates for the poor physical overlap.

A figure of merit for remote pairs. Besides, the field enhancement (Figure 1d, right panels) could provide evidence for evaluating the capability of harvesting light and enhancing the light-matter interaction. NCoM has the most prominent field enhancement around 117 folds while the largest of NSoM and NWoM are 53 and 76 folds. One could roughly estimate the remote pairs by combining the field enhancement and the SPPs conversion efficiency, i.e., roughly |**E**/**E**_0_|^4^ *η* ^2^. Here, the receiving and transmitting processes altogether follow |**E**/**E**_0_|^4^. The efficiency *η* ^2^ accounts for the conversion of launching SPPs and coupling SPPs into the transmitting antenna. Note that the SPPs would decay at the same rate related to the metal film absorption; this part could be omitted in the comparison among three configurations. Having a much larger |**E**/**E**_0_|^4^ and decent efficiency *η*, the NCoM pair should be well capable as a remote configuration.

If we elaborate more on the evaluation of the remote configurations, a remote figure of merit FOM* could be defined (detail see Methods). As shown in Figure 1e and f, we could define and calculate the remote Purcell factor (*F*_p_*) where we put a dipole at the receiving antenna and calculated the radiation from the transmitting antenna 5 μm away. (detail see Methods) Figure 1e1 and f1 compared the remote *F*_p_* of NCoM and NSoM, showing *F*_p_* of the NCoM is more than 10 times larger than NSoM. The *E*-field of launched SPPs in Figure 1e3 and f3 (sharing the same colorbar) visualized that NCoM has a much greater portion of in-coupled SPPs than NSoM, in correspondence to the factor *F*_p_*. This remote *F*_p_* has taken the SPPs launching and remote photon radiation into account. By combining with the electric field at the receiving antenna, mimicking the definition of local SEF, we arrive at a remote figure of merit, FOM* = |**E**/**E**_0_|^2^ × *F*_p_* (details see Methods). Therefore, due to a prominent *E-*field enhancement and remote *F*_p_*, NCoM pairs would be chosen as a demonstration in the following experiments. Nonetheless, all three structures are basic building blocks for remote sensing, though with some differences in efficiencies and application scenarios (also find comparisons in Figure S7 in SI).

**Figure 2:**
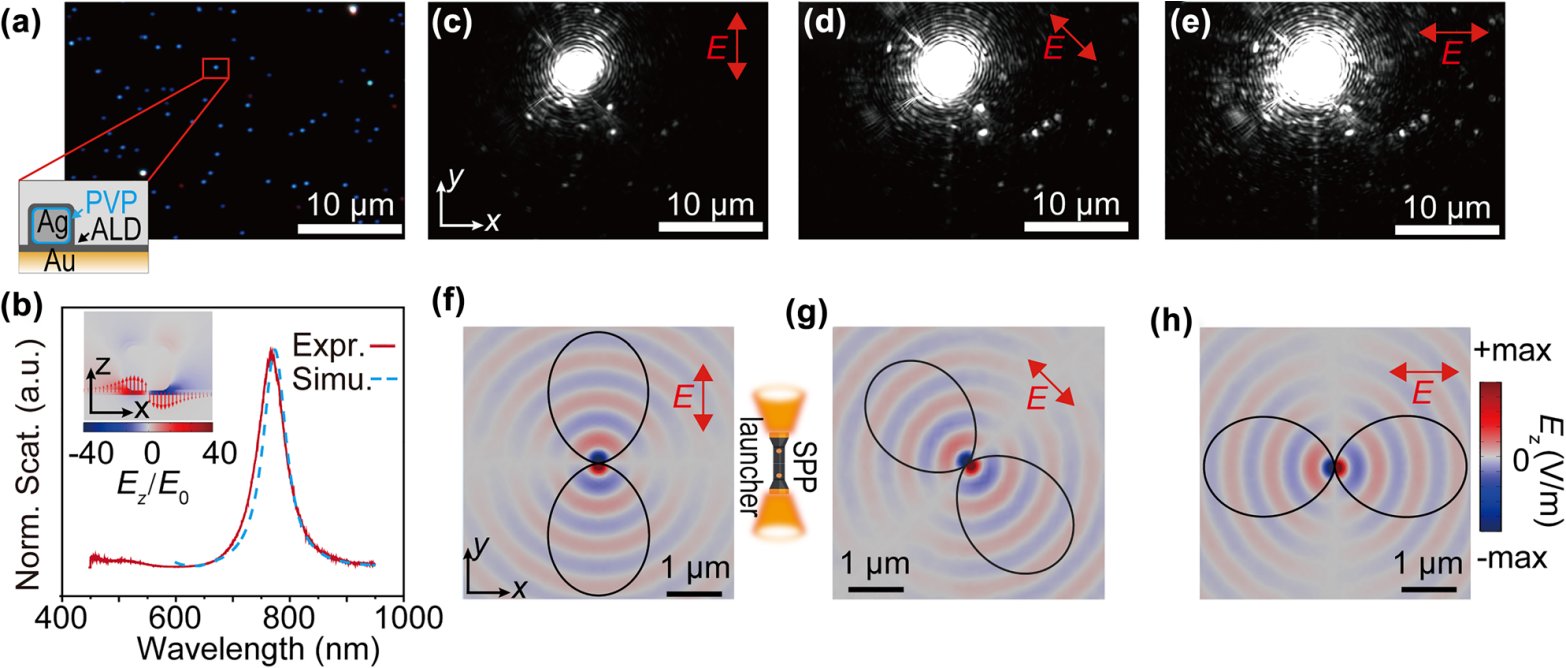
SPPs traveling between NCoM pairs and remotely lightening the transmitting antennas. Dark-field image (a) and scattering spectrum (b) of the NCoMs. The inset of (b) shows the local field in the NCoM cavity. (c–e) are the images of NCoM excited by the laser on resonance (770 nm). Many NCoMs far away from the laser show bright emissions, revealing that they were excited by SPPs. (f–h) are the simulated *E*_z_ field of the SPPs propagating on the surface launched by the NCoM, with different *E-*polarization. Black curves are the far-field diagram of the SPPs.

Remote transmission of light. Before we investigate remote spectroscopies, we start by elaborating on the fundamental properties of remote excitation. Here we prepared NCoM sample with *s*_11_ resonance around 770 nm (sample preparations see Methods). The dark-field image (Figure 2a) ensures proper dispersion of the nanocubes on the substrate, with the experimental and simulated scattering spectra (Figure 2b) showing resonance at 770 nm. The alumina spacer produced by atomic layer deposition (ALD) on the gold film is 7 nm, and the 100 nm nanocube (bought from nanoComposix) was assumed to be covered by 3 nm PVP. The whole NCoM was sealed by 5 nm alumina deposited with ALD (as shown in Figure 2a inset).

To verify the remote transmission characteristics of the NCoM pairs, a laser with a wavelength matching the resonance of NCoM is focused on the sample. As a result, different parts of the NCoM sample were lightened, intuitively visualizing the far-field diagram of the launched SPPs. All the bright spots in Figure 2c–e could be corresponding to the dark field image (Figure 2a). Then we rotated the electric field polarization by 90° (Figure 2c–e), from *y*-axis (Figure 2c) to *x*-axis (Figure 2e). It can be seen that the propagation direction of SPPs has obvious polarization dependence. We calculated the *E*_z_ field of the SPPs launched by the fixed NCoM illuminated by different polarized plane waves (incident polarization marked in the insets of Figure 2f–h). From the *E*_z_ field, we observed that the launched SPPs direction in the simulation corresponded to the experiments. And the far-field diagrams of the SPPs (Figure 2f–h, patterns in black) indicate that the launching direction of the SPPs is along with the incident *E*-polarization (see Methods for details of the far-field diagram).

The reason for the coincidence between SPPs far field and the incident polarization could be revealed by understanding the *s*_11_ mode of the NCoM (inset of Figure 2b). The NCoM could be understood as a truncated metal-insulator-metal (MIM) waveguide [8] with a finite length determined by the size of the cube together with extra reflection phases around the edges [39, 40]. Let’s take *x*-polarized incident light for instance (Figure 2h and inset of Figure 2b). Confined by the finite size of the cube, the MIM mode would reflect back and forth around the edges of the cube due to the impedance mismatch, which forms a group of standing waves at the *x*-direction. The lowest-order cavity is the *s*_11_ mode (inset of Figure 2b) [39, 40]. As a result, when this plasmonic waveguide mode reflects near the edge, a portion of the energy would leak out and form the SPPs on the film. This explains the reason why the *x*-polarized *s*_11_ mode gives rise to the *x*-directional SPPs launching. This NCoM SPPs launcher works as a nano “torch” (schematic inset of Figure 2f) that lightens the directions following the polarization.

**Figure 3:**
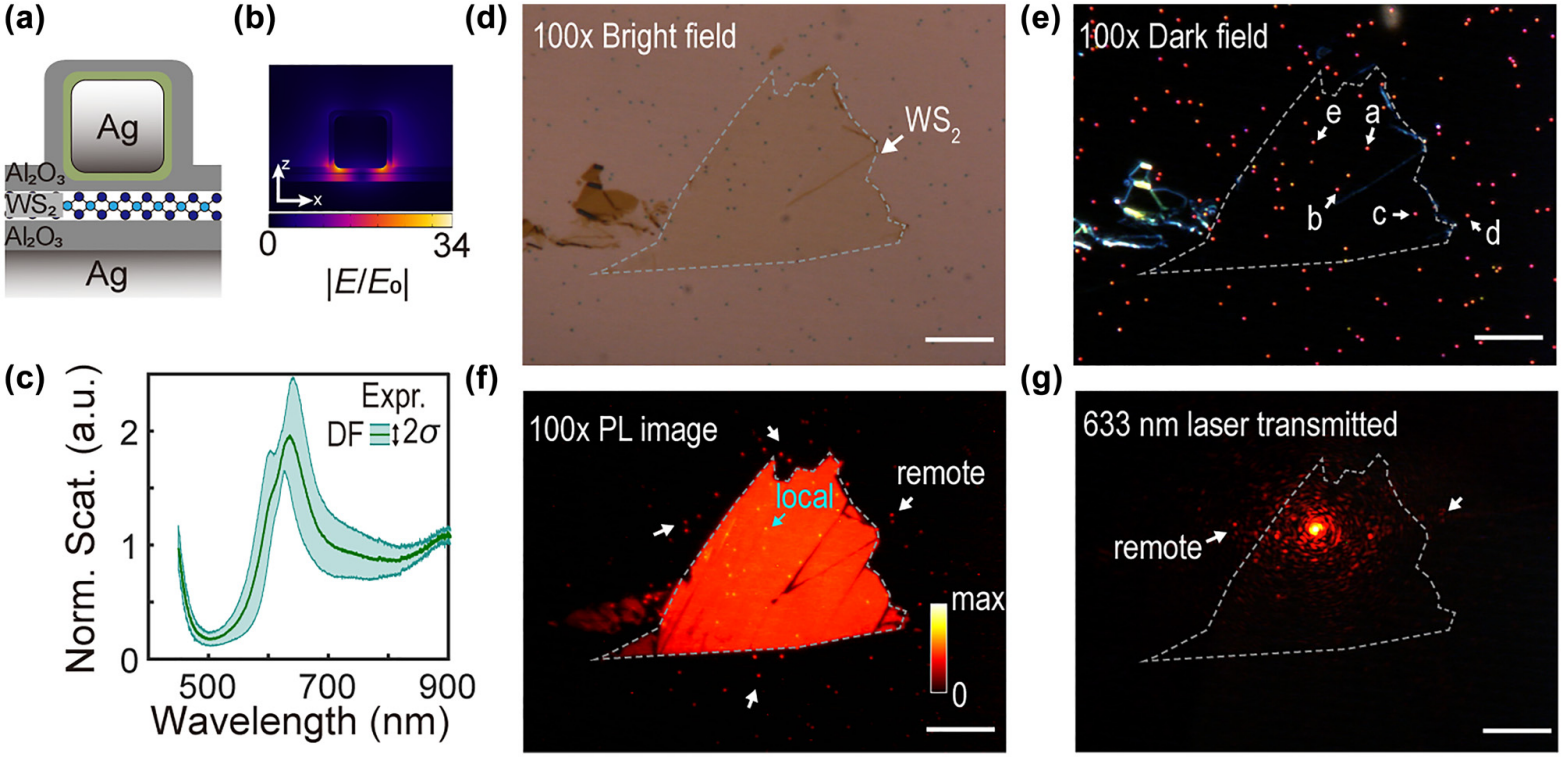
Demonstration of remote properties on NCoM-WS_2_ hybrids. (a) Schematic of NCoM-WS_2_ hybrid composed of Ag nanocube and Ag film, spaced by alumina layers and monolayer WS_2_. (b) The field enhancements of the hybrid system. The plane shown is the central cross-section of *xz*-plane. (c) The average dark-field scattering over 10 samples with green shades indicating the standard deviation. Bright field (d) and dark field (e) images of the hybrid system. (f) PL image excited by a mercury lamp. (g) Optical images of SPPs launched and scattered out by the NCoMs. Receiving NCoM was locally excited by a 633 nm laser. Gray dashed lines in (d–g) mark the edges of monolayer WS_2_. Scalebars represent 10 μm. (f) and (g) share a same colorbar.

Remote SEF and SERS of the NCoM-WS_2_ hybrid. To demonstrate the performances of remote SEF and SERS in these NCoM pairs, we built up a NCoM-WS_2_ hybrid system as Figure 3a (see Methods). These nanocavity-transition metal dichalcogenides complex nanostructures were intensively studied for understanding the light–matter interaction in scattering, Raman and PL [11, 13], [14], [15]. An ultrasmooth Ag film was deposited by 11 nm alumina, monolayer WS_2_, and 4 nm alumina, in sequence. A 75 nm Ag nanocube covered by around 3 nm PVP (bought from nanoComposix) was deposited onto the substrate and sealed with 5 nm alumina capping on top (bright-field and dark-field image in Figure 3d and e). This large gap thickness (∼18 nm) is for tuning the *s*_11_ plasmons to match the WS_2_ transition around 615 nm. Note that the geometric parameters for this resonance are not unique, and one could further optimize the nanoparticle sizes, materials, spacer thickness, etc., for better enhancement and emission efficiencies [41]. It is worth mentioning that NCoM-WS_2_ hybrids are possible to induce spin-forbidden dark excitons at room temperature [42, 43]. Nevertheless, in our experiment, we did not conduct extra processing on the hybrids, so the bright excitons are dominant. Besides, our PL with 532 nm laser excitation (SI Figure S1) further convinced us that we have a neglectable contribution from dark excitons.

In Figure 3c, the experimental scattering averaged from NCoM-WS_2_ hybrids confirmed the near resonance between plasmons and WS_2_ excitons. We could observe a weak coupling between bright plasmons and WS_2_ excitons from the scattering spectrum, where a slight Fano dip and a shoulder were formed (green curves). We could also find the trace of the plasmon-exciton weak coupling in the standard deviation (green shades and boundaries) and dark field scattering from various NCoMs (see Figure S3 in SI S2). Due to the large nanogap, the electric field enhancement (Figure 3b) would be smaller than that of 5 nm (Figure 1d) and this weak enhancement leads to the plasmon-exciton weak coupling. However, we still could observe prominent photoluminescence (PL) from image in Figure 3f.

As the sample was excited by a mercury lamp for a PL image (Figure 3f), we observed the monolayer WS_2_ having a homogeneous PL emission, and there were lots of separated bright spots indicating PL emission from the NCoMs. The correspondence between such spots in the PL image (Figure 3f) and the dark field (Figure 3e) ensures that the PL originated from the NCoM nanoantennas. Taking a closer look at the PL image, we would find many brighter spots on the WS_2_ indicating a locally enhanced PL emission (local SEF), while many less-bright spots off the WS_2_ also harbor considerable PL emission (comparable to the bare WS_2_ PL background). These off-WS_2_ NCoMs proved that their PL emissions are remote: the PL from the on-WS_2_ NCoM was delivered to the off-WS_2_ NCoM via launching the SPPs carrying the information of PL. By directly exciting the NCoM on the WS_2_ with a near-resonant laser of 633 nm, we could observe the SPPs outcoupled from remote NCoMs in Figure 3g.

**Figure 4:**
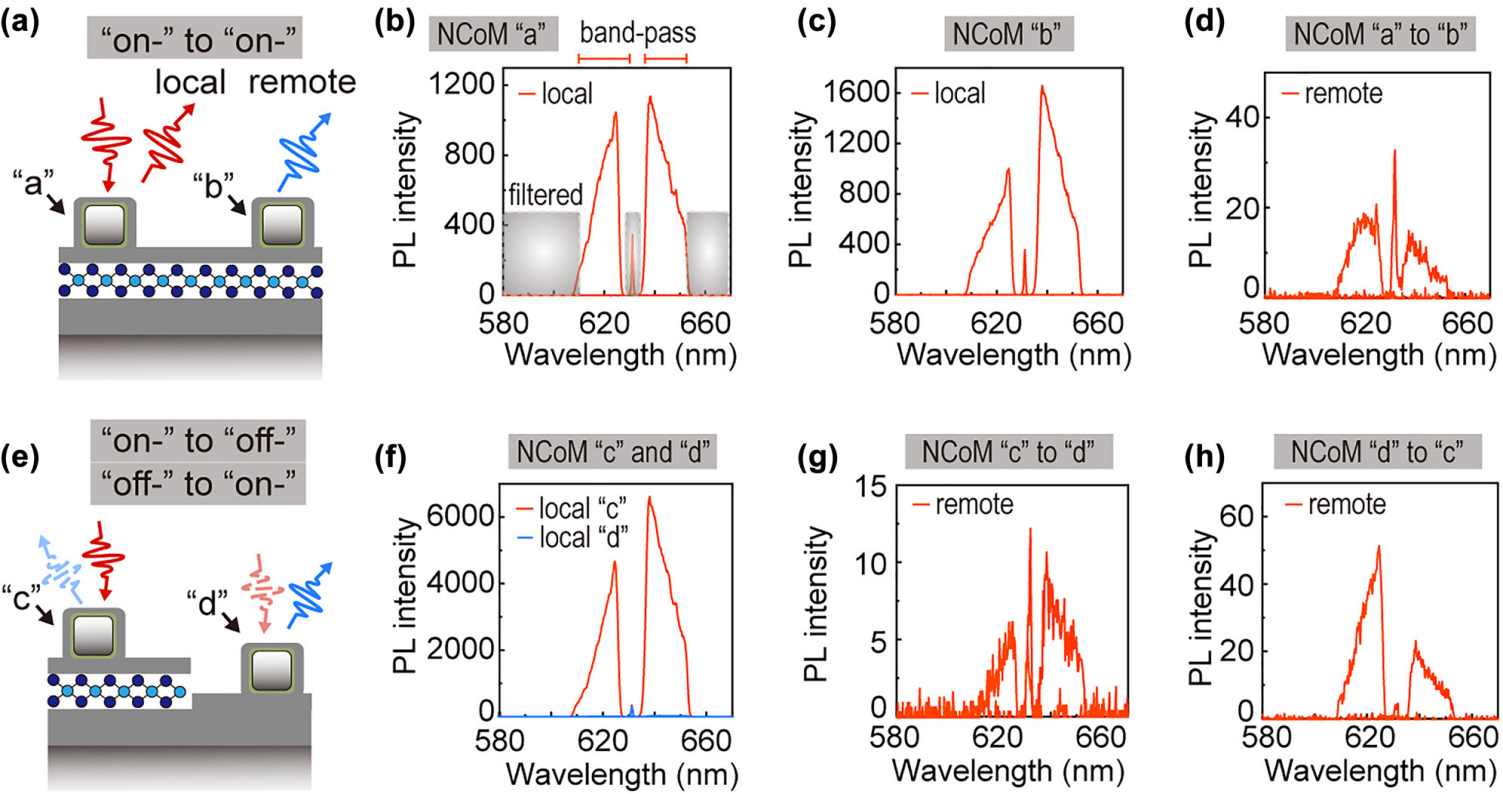
Remote WS_2_ SEF realized by NCoM pairs. (a–d) show the local (b, c) and remote (d) PL spectra of the NCoM pair whose receiving and transmitting antennas were all on WS_2_, schematic in (a). (e–h) Show the local (f) and remote (g, h) PL spectra of the NCoM pair whose receiving or transmitting antenna was off WS_2_, schematic in (e). Schematics are not to scale.

**Figure 5:**
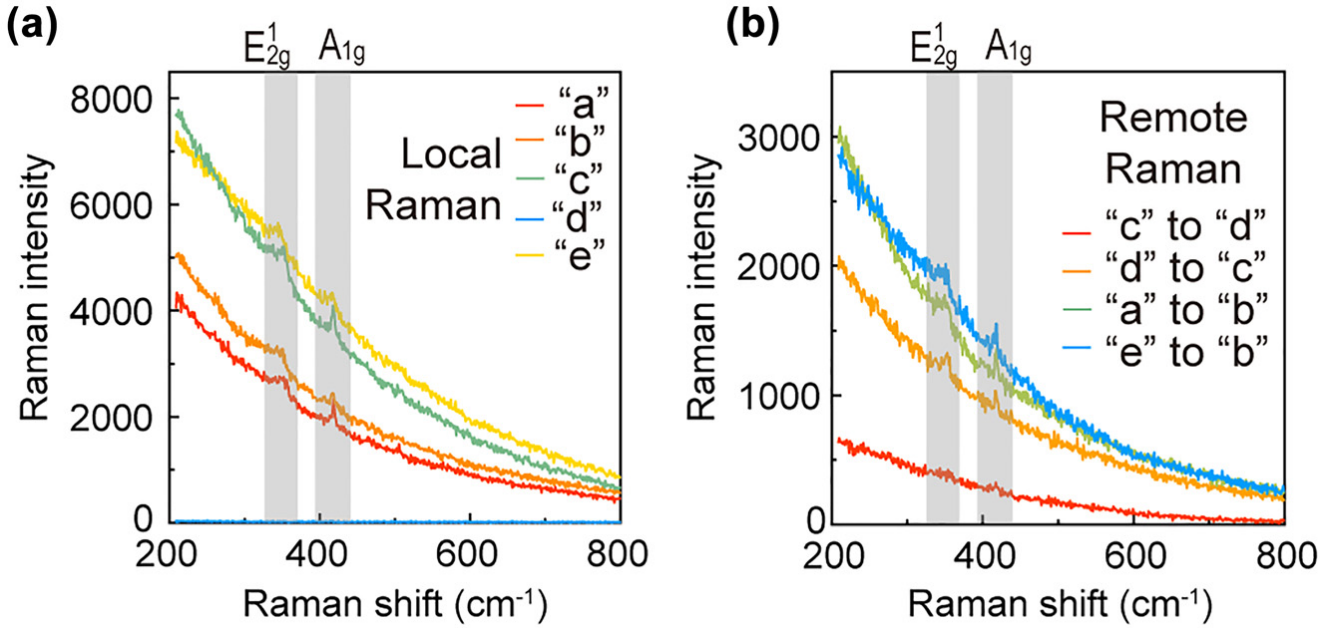
Remote WS_2_ SERS realized by NCoM pairs. Local (a) and remote (b) Raman scattering spectra from NCoM pairs. Grey shades mark two prominent Raman modes: A_1g_ (∼420 cm^−1^) and ${\text{E}}_{2\text{g}}^{1}$ (∼350 cm^−1^).

Based on this NCoM-WS_2_ hybrid and receiving-transmitting-separated functions, we could further illustrate how these NCoM pairs work in remote SERS and SEF. NCoMs labeled as “a”-“e” (in Figure 3e) were involved in our demonstration of remote SERS and SEF experiments in Figures 4 and 5. Massive measurements over different NCoM pairs could be found in SI S2 Figure S2–4. The excitation-collection-separated remote configurations include three situations: (1) NCoM pairs are all on the monolayer WS_2_ (“on-” to “on-”), e.g., NCoM “a” to “b”, (2) the receiving NCoM is on WS_2_ yet the transmitting is off WS_2_ (“on-” to “off-”), e.g., NCoM “c” to “d”, (3) the receiving NCoM is off WS_2_ yet the transmitting is on WS_2_ (“off-” to “on-”), e.g., NCoM “d” to “c”. All situations were investigated and proved to support remote spectroscopies with considerable SERS and SEF enhancements.

For NCoM pair with receiving (“a” in Figure 3e) and transmitting (“b” in Figure 3e) antennas all on WS_2_ (Figure 4a–d), separated by around 7.6 μm, we could investigate the PL on each functional part (Figure 4b and c) and their remote PL excited at “a” yet collected at “b” (Figure 4d). Here we chose 633 nm excitation because the 633 nm is near the resonance of the receiving NCoM (Figure 3c) which would guarantee an efficient SPP conversion. We also perform PL excited by 532 nm laser, which also shows remote behaviors (see SI Figure S1). Because 532 nm is off-resonance, it could hardly excite SPPs to power the SEF and SERS at the transmitting antenna. The PL acquired from the remote end comes from the WS_2_ fluorescence (∼620 nm) generated at the receiving antenna, which could be efficiently converted into SPPs due to the resonance match.

Because the incident 633 nm laser somewhat overlaps with the WS_2_ fluorescence, we used a tunable band-pass filter to measure the blue- and red-side of the PL spectra in sequence, and then put them in the same PL spectrum (see Methods). As schematically shown in Figure 4a, we first examine and compare the local and remote PL (Figure 4b–d) from two on-WS_2_ NCoM labeled as “a” and “b” in Figure 3e. They are all on monolayer WS_2_, proved by the dark-field and PL image (Figure 3f). In Figure 4b and c, the local PL of the receiving (NCoM “a”) and transmitting (NCoM “b”) antennas show great PL intensity of WS_2_ enhanced by the NCoM. The remote spectroscopy (Figure 4d) also managed to evidently capture the same PL signature from the transmitting antenna, with around 50 times less intensity.

Similarly, we could investigate the other two situations where either the receiving or the transmitting antenna is off the WS_2_ layer. From the dark-field and PL image (Figure 3e and f) we could verify that the NCoM “c” is on WS_2_ yet the NCoM “d” is off WS_2_ (as sketched in Figure 4e). We could either excite “c” or “d” to study the remote PL from “d” or “c”, respectively. Local PL (Figure 4f) ensures that there are no signals from the off-WS_2_ antenna “d” (blue), while the on-WS_2_ antenna “c” has tremendous PL intensity (red). Both situations, i.e., either serve as receiving or transmitting antennas; present evident remote SEF (Figure 4g and h) though with different intensities. The inside physics could be different, (1) from “c” to “d”, the SPPs have already carried the PL signals, and the transmitting antenna barely broadcast it out; (2) from “d” to “c”, the receiving antenna “d” harvests and converts the free-space photons into SPPs on the film. The SPPs excite the on-WS_2_ transmitting antenna “c” and drive the plasmon-enhancing PL. The transmitting antenna “c” converts the near-field modes into photons carrying the PL signals.

The latter (“off-” to “on-”) case (Figure 4h) obviously has more intense and less-noisy spectra than the former (“on-” to “off-”) case (Figure 4g). This is reproducible and the relationship could be found in statistics (Figure S4 in SI). The physics for this difference lies in the excitation and function methods mentioned above. For case (1) (“on-” to “off-”), the PL from the receiving antenna couples into SPPs which could be reabsorbed by the WS_2_ due to energy match. For case (2) (“off-” to “on-”), the 633 nm laser was coupled into SPPs and this energy is detuned from the WS_2_ excitons transition, which could be less absorbed by the WS_2_ due to the energy mismatch. Therefore, the “off-” to “on-” case is more efficient than the “on-” to “off-” case. Anyhow, these two cases all suffer from prominent losses by the light scattering at the boundary of WS_2_. Therefore, the “on-” to “on-” case (Figure 4a–d) has the best efficiency revealed by statistics (Figure S4 in SI). In Figure S2–4 in SI S2, we have done massive measurements over 26 NCoM pairs and compared their local and remote PL (efficiency *β* = *I*_rem_/*I*_loc_) statistically, proving the specific and characteristic data shown in Figure 4 is reliable. The simulated results comparing remote and local Purcell factors (*β*_cal_ = *F*_p_*/*F*_p_) accord well with the experimental *β* (Figure S4).

Lastly, in Figure 5, we could demonstrate the proof-of-principle remote SERS on these NCoM pairs. Local Raman scatterings were measured on five NCoMs “a” to “e” labeled in Figure 3e. Four of them are on-WS_2_ and one is off-WS_2_. As shown in Figure 5a, except for the off-WS_2_ NCoM “d”, every NCoM shows prominent Raman signals overlapping on the broad PL background. More evident Raman could be observed with 532 nm excitation due to the elimination of the overwhelming PL background (as shown in SI Figure S1a–c). But 532 nm excitation shows no remote SERS due to the failure of exciting SPPs because of the resonance mismatch. Two evident Raman modes were observed at 420 cm^−1^ (labeled as A_1g_ mode) and 350 cm^−1^ ( ${\text{E}}_{2\text{g}}^{1}$ + 2LA mode), labeled as ${\text{E}}_{2\text{g}}^{1}$ hereafter for simplicity [44]. Remote SERS spectra among such antennas are shown in Figure 5b. Except for the “c” to “d” case with high noise, all pairs had successfully detected the target Raman signals (A_1g_ and ${\text{E}}_{2\text{g}}^{1}$ ). This low signal-to-noise ratio remote SERS presented by the “c” to “d” corresponds to their noisy remote PL spectrum (Figure 4g). Therefore, the different ways of information delivery will affect the performance of the nanoantenna pairs.

The NCoM-WS_2_ hybrid has been proven to be capable of remote SEF and SERS. Moreover, these NPoM-family configurations are easy to be compatible with two-dimensional materials, quantum dots, molecules, etc., manifesting their strong compatibility. On the one hand, an individual receiving nanoantenna can launch SPPs and generate remote signals at several transmitting nanoantennas. The SPPs could serve as a non-interfering information carrier. On the other hand, for remote spectroscopies, using the vertical electric field of SPPs to efficiently excite and drive the SERS/SEF in the transmitting antenna for remote spectral signals is also a very effective plan.

In reality, the NPoM pairs were prepared by drop-casting nanoparticles (nanospheres, nanocubes, and nanowires) onto a designed substrate. The nanoparticle sizes have variations, and the orientations of the nanoparticles would be random. The orientation of the nanocubes could be an issue but is proven not to affect the remote sensing performance (Figure S5 in SI). But the size differences are proven to be significant where two identical nanoparticles with the same resonances would obviously have higher efficiency. As discussed and shown in SI Figure S8, we tuned the size of the transmitting antenna from 60 to 100 nm, yet fixed the receiving antenna to be 80 nm (same as in Figure 1). We could see the maximum of the remote Purcell *F*_p_* would lift and drop, due to the match and mismatch between the resonances in the two NCoM antennas.

Moreover, when performing the experiments, we could frequently find extra nanoparticles situated on the path between the remote pairs (e.g., “a” to “b” in Figure 3e). But we could still acquire decent spectra remotely. To justify this, we calculated and compared the remote Purcell with and without this extra nanocube. In Figure S6 in SI, we found that both the spectra and electric fields were weakly perturbed by this “obstacle” nanoparticle.

## 3 Conclusions

In summary, we have demonstrated that NPoM pairs (NCoMs, NSoMs, NWoMs, etc.) could be ideal candidates for remote spectroscopies, and systematically delivered proof-of-principle demonstrations for their usages in remote SEF and SERS. We theoretically calculated the fundamental optical properties of these pairs and defined a figure of merit for remote configurations. NCoM pairs seem to have superior performance when they are compatible with two-dimensional materials, and we experimentally demonstrate the remote SERS and SEF conducted by NCoM pairs, which have considerable remote SEF and SERS enhancements. This work comprehensively compares and weighs different kinds of NPoM antennas, giving guidelines and enriching the toolbox for on-chip remote spectroscopies. Also, these kinds of nanoconfigurations have great potential for application in precise spectral characterization, early diagnosis, and monitoring in modern biomedicine, biphotonic detection, and wireless optical communication.

## 4 Methods

Efficiencies of scattering and SPPs. The efficiencies *Q*_scat_ and *Q*_spp_ follow the well-known definition of the absorption, and scattering of light by nanoparticles [35]. They clearly relate to the cross-sections of the light converted into scattered photons *C*_scat_ and SPPs *C*_spp_, which intuitively answers the question: when the light passes by the nanostructure, how large is the area affected by the nanostructure? The efficiencies obtained by normalizing these cross-sections with individual physical cross-sectional areas offer opportunities for a fair comparison between different structures. And considering an incident light beam with a finite waist (diameter *r*_0_ for example), we could roughly estimate the in-coupled efficiencies *η* by comparing the cross-sections with the incident beam waist, $\eta =Q_{\text{spp}}\times S_{0}/\pi {r_{0}}^{2}$ (3D case), *η* = *Q*_spp_ × *D*_0_/2*r*_0_ (2D case, only for NWoM, *D*_0_ is the nanowire’s diameter). The calculation of scattering and SPPs efficiencies *Q*_scat_ = *W*_scat_/(*I*_0_ × *S*_a_), and *Q*_spp_ = *W*_spp_/(*I*_0_ × *S*_a_) were performed via near-to-far-field transformation using an open-source package by Yang et al. [30]

In the full-wave simulation package COMSOL Multiphysics 5.2a, 3D geometries were built for NCoM and NSoM, and 2D geometry was built for NWoM. Two steps were arranged: the first step is for calculating the background field without scatters, while the second step is for calculating the scattered field from the scatters. (1) *Scattering energy:* we did a near-to-far-field transformation and calculated the Poynting vector **S**_scat_ and its surface integral *W*_scat_ = *∫* **S**_scat_ ⋅ **n** *dA* in the far field. It would only consist of the scattering energy, unlike the Poynting vector in the near field which may be mixed with SPPs components. (2) *SPPs energy:* SPPs energy could also be retrieved from near-to-far-field transformation following ref. [30] where the Lorentz equivalence principle and reciprocity theorem were applied. The permittivity of gold and silver was taken from Johnson et al. [45]. The Refractive indexes of alumina and PVP were 1.5. The permittivity of WS_2_ follows $\varepsilon \left(\omega \right)=\varepsilon_{\infty }-\frac{f{\omega_{\text{e}}}^{2}}{\omega^{2}-{\omega_{\text{e}}}^{2}+i\Gamma_{\text{e}}\omega }$ , where *ε*_∞_ = 16, *f* = 0.05, *ω*_e_ = 2.0496 eV, and Γ_e_ = 22 meV are the high frequency dielectric contribution, oscillator strength, exciton transition frequency, and exciton linewidth, respectively.

Remote Purcell factor *F*_p_* and figure of merit FOM*. Remote Purcell factor *F*_p_* was defined as a part of the figure of merit FOM* to evaluate the performance of remote spectroscopies. The whole remote spectroscopy could be divided into three separated but linked processes, (1) light harvesting from the free-space photon into near-field modes by receiving antenna, which could be qualified by the enhancement factor |**E**/**E**_0_|^2^; (2) near-field excitations under receiving antennas leaking out of the nanocavity and transformed into SPPs on the metal film; (3) SPPs exciting the transmitting antenna and outcoupling to the far field in the form of free-space photons. The last two processes could be overall represented by defining a “remote Purcell factor”, *F*_p_* = *P*_tran_/*P*_0_. Here, a point dipole was inserted under the receiving antenna, and the *P*_tran_ means the power of the radiation from the transmitting antenna. Dipole radiation power in the vacuum $P_{0}=\frac{|p{|}^{2}}{4\pi \varepsilon_{0}\varepsilon }\frac{n^{3}\omega^{4}}{3c^{3}}$ . The figure of merit FOM* of the remote systems could be defined by combining the aforementioned two factors, which reads FOM* = |**E**/**E**_0_|^2^ × *F*_p_*. One may find this FOM* is intuitively suitable for the systems where the analytes are placed in the receiving antenna. We need to emphasize that due to the antenna’s identity and reciprocity, this FOM* could also account for the performance of analytes placed in the transmitting antenna.

Sample preparation. The NCoM samples were prepared by simply drop-casting nanocubes onto the prepared substrates, and sealed with alumina layers by ALD at 120 °C. The substrates used in our paper were: (1) barely alumina layers with ultrasmooth Au film (Figure 2); (2) alumina layers, monolayer WS_2_, with ultrasmooth Ag film (Figure 3). The ultrasmooth Au films and Ag films were prepared following template-stripped method [46]. The monolayer WS_2_ was mechanically exfoliated onto the film. Through the CCD image contrast, the monolayer WS_2_ can be identified. PL and Raman spectra were also used to characterize the sample.

Spectroscopy. The dark-field scattering spectroscopy was utilized to characterize the plasmon resonance of the NPoM. A halogen lamp illuminated the samples and then the scattered light was collected with an Olympus objective (100×, NA = 0.8). Then the signal was directed to a Renishaw inVia spectrometer equipped with an air-cooled CCD and a 300 lines/mm grating blazed at 1 μm. The PL spectra were obtained through the same objective and spectrometer, only substituting a 633 nm laser for the halogen lamp. The collection signal passed through the tunable bandpass filter (Semrock TBP01 697) to suitable angles to, respectively, capture the blue- and red-side of the PL spectra. The PL image was acquired by exciting the sample with a mercury lamp, after passing through a 460–490 nm band-pass filter. Then the signal passed through a 520 nm long-pass filter before being collected by a CCD camera (Tucsen, TCH-1.4CICE).

## Supplementary Material

Supplementary Material Details
