# Supplementary material for: Nanoparticle-on-mirror pairs: building blocks for remote spectroscopies
Source: Nanophotonics. 2022 Oct 27;11(22):5153–63. doi: 10.1515/nanoph-2022-0521 (PMC11501606; doi:10.1515/nanoph-2022-0521)
Supplement: Supplementary file 1 — Supplementary Material Details [file j_nanoph-2022-0521_suppl.docx]

# Supporting Information for “Nanoparticle-on-mirror pairs: building blocks for remote spectroscopies”

*Huatian Hu,* ^†,‡,#^ *Yuhao Xu,* ^⁋,#^ *Zhiwei Hu,* ^‡^ *Bowen Kang,*^§^ *Zhenglong Zhang,*^§^ *Jiawei Sun,*^,^*^†^ *Yang Li*^,^* ^†^ *and Hongxing Xu,*^,^* ^†^*^,^* ^⁋^

^†^School of Electronics and Information Engineering, Shenzhen University, Shenzhen 518060, China.

^‡^Hubei Key Laboratory of Optical Information and Pattern Recognition, Wuhan Institute of Technology, Wuhan 430205, China

^⁋^School of Physics and Technology, Center for Nanoscience and Nanotechnology, and Key Laboratory of Artificial Micro- and Nano-structures of Ministry of Education, Wuhan University, Wuhan 430072, China.

^§^School of Physics and Information Technology, Shaanxi Normal University, Xi’an, China

**Corresponding Author**

*E-mail: jwsun@szu.edu.cn

*E-mail: yang.li@szu.edu.cn

*E-mail: [hxxu@whu.edu.cn](mailto:hxxu@whu.edu.cn). Phone: +8627 68752253.

**S1. Local and Remote SERS and SEF excited by 532 nm laser**

Here we examine the local and remote SERS and SEF spectra of NCoM pairs constituted by “a” and “b”, “c” and “d” NCoMs, respectively. See “a” – “d” labels in main text Fig. 3e. The receiving antenna was driven by 532 nm laser, and the remote SERS and SEF spectra overlap with each other. From Fig. S1a-c, we could see both Raman and PL signals overlapping with each other when detecting locally. However, when we remotely excited the NCoM pairs (separated excitation and collection points), we would only observe PL signals Fig. S1d, e without the remote Raman signals. This is because either the 532 nm excitation or WS_2_ Raman modes excited by the 532 nm laser would all be far away from the NCoM’s plasmon resonances, which makes the Raman modes hardly be transported via the SPPs. And due to the poor excitation rate, there would hardly be 532 nm SPPs on the film, let alone driving the transmitting antenna by SPPs. The remote PL acquired at the transmitting antennas is all from the PL generated at the receiving antenna. Due to the matched resonances between WS_2_ exciton and NCoM plasmons, this PL (~ 620 nm) could be efficiently converted into the form of SPPs and emitted out from the transmitting antenna.


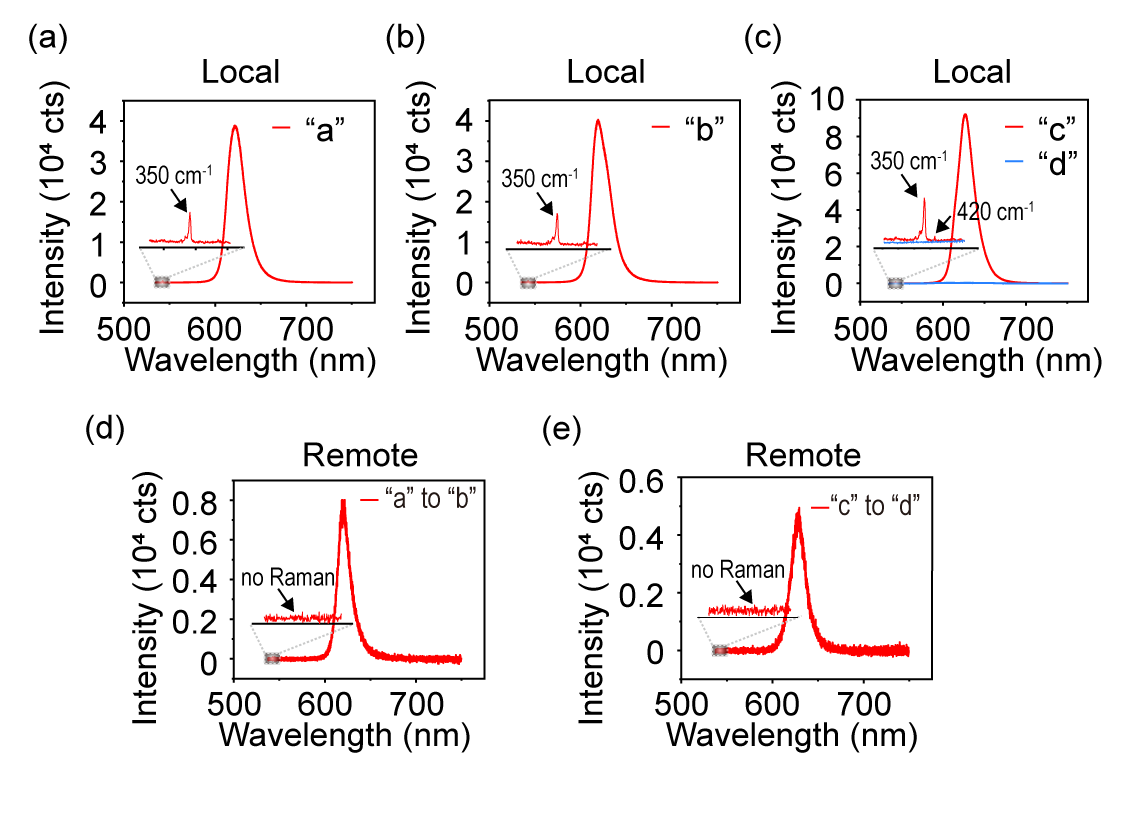


**Figure S1**: Local (a-c) and remote (d,e) spectra of the NCoM pairs. Raman and PL overlap with each other. The inset is the enlarged area magnifying the Raman spectra for clarity.

**S2. Remote PL spectroscopy of various NCoM pairs**

Here we performed massive measurements over 26 NCoM pairs containing all three cases: remote PL from “on- to on-”, “on- to off-”, and “off- to on-” cases defined in the main text. Here, for simplicity, we measured the remote PL using a bandpass filter (see Methods in the main text) and only demonstrated the long pass spectra (Fig.S2). We could define an efficiency parameter *β* = *I*_rem_/*I*_loc_ from the experiments that could reflect the efficiency of the remote system, where *I*_rem­_ is the intensity (counts) of the remote PL and *I*_loc_ is the intensity (counts) of the local PL. For instance, when we evaluate the performance of the remote PL from NCoM No.1 to No.2. We could excite the No.1 with a 633 nm laser and collect the remote PL *I*_rem_ at NCoM No.2, then divide by the local PL *I*_loc_ (excited, collected at the same place).


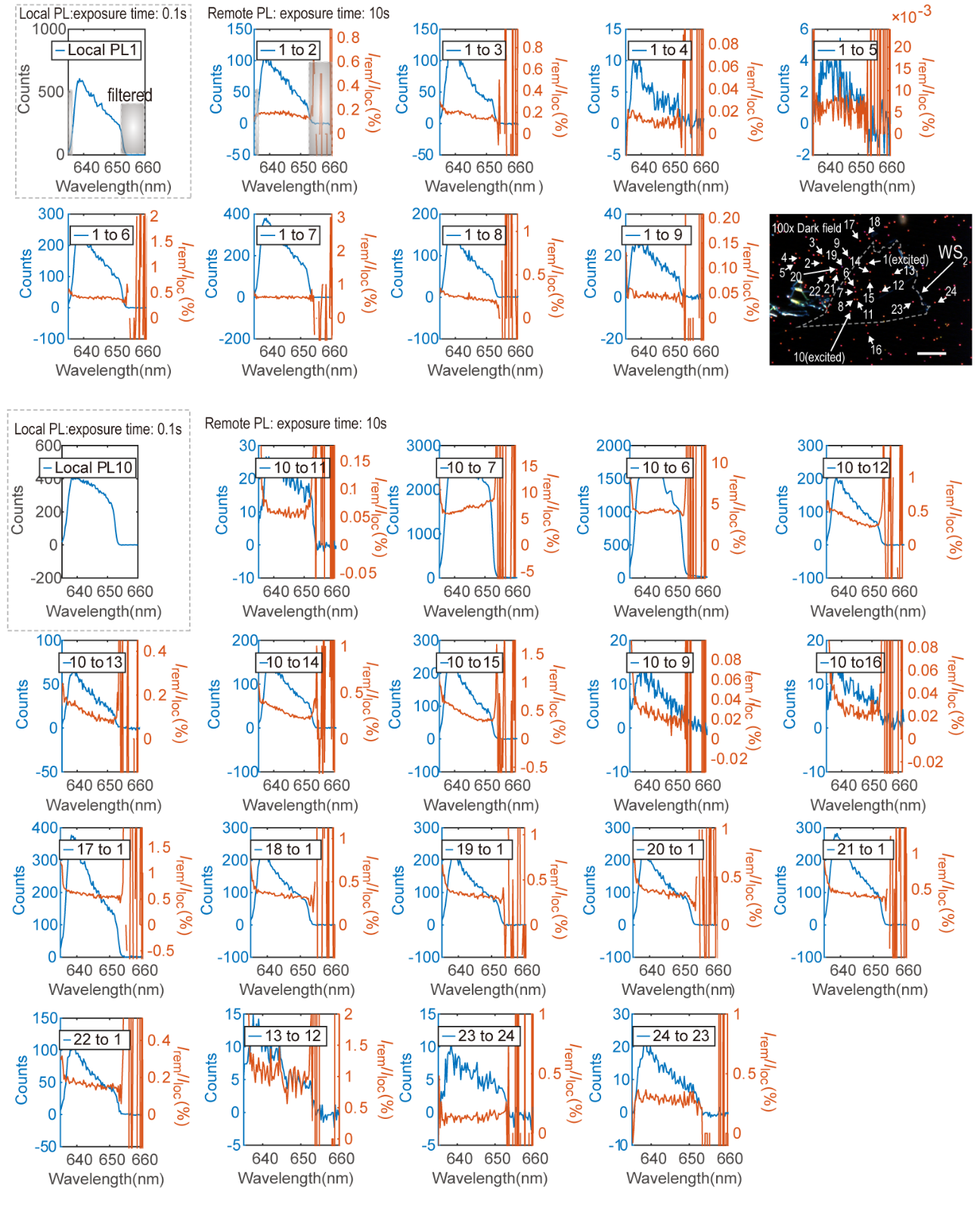


**Figure S2**: Data from 26 different NCoM pairs. Blue lines indicate the PL counts (exposure time: local PL - 0.1s, remote PL - 10s), and the red lines indicate the efficiency defined by the intensity of remote PL divided by local PL, namely *I*_rem_/*I*_loc_. The 100x dark field image marks the sequence number of each nanoparticle we measured in the experiment.

The corresponding dark field scattering data were shown in Fig. S3. From Fig.S3 we could observe weak coupling between plasmon and excitons (Fano dips) from various NCoMs, e.g., No.6, 7, 8, 11. These scattering spectra indicate that the exciton resonances and plasmons are basically near-resonant. And a decent plasmon enhancement could be expected.


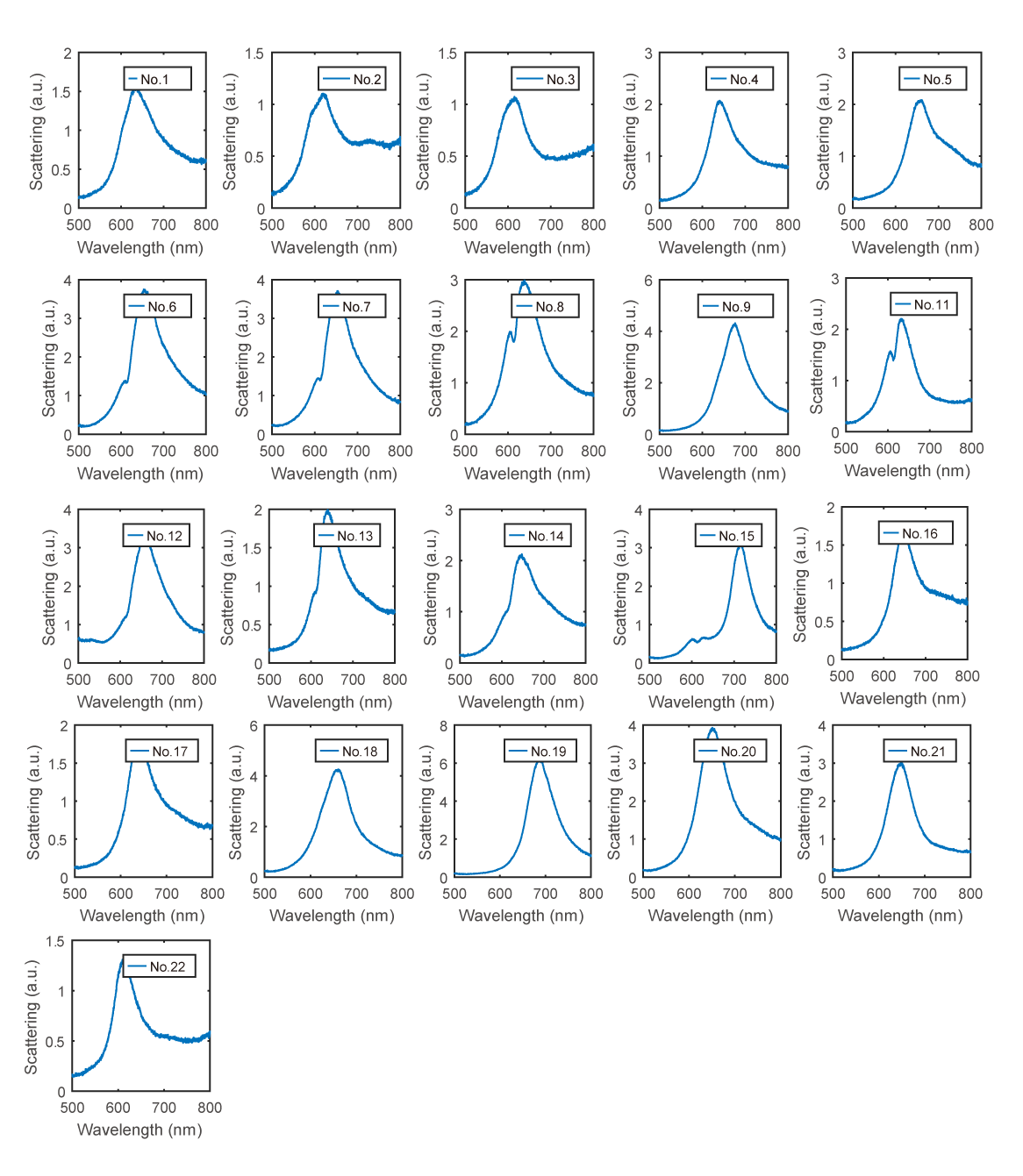


**Figure S3**: Dark-field scattering of the NCoMs used in Fig.S2. The legend indicates the sequence number of the nanoparticle we used.

After analyzing the efficiency *β* = *I*_rem_/*I*_loc_ (red lines of Fig. S2) and reorganizing the data with the distance between NCoMs, we obtained a clear decay manner upon the increasing distance (scatters in Fig. S4). As a comparison, we could calculate the theoretical efficiency *β*_cal_ = *F*_p_*/ *F*_p_ defined as the remote Purcell factor *F*_p_* divided by the local Purcell factor *F*_p_. The theoretical results (red line with black dots) accord with the experimental results in Fig. S4.

In addition, we could find that though with considerable fluctuation, we still observed the efficiency of the “on-on” case (gray scatters) is larger than “off-on” case (blue scatters). The “on-off” case (red scatters) has the least efficiency. It is straightforward that the “on-on” case has the highest efficiency due to the absence of boundary scattering from the WS_2_. On the opposite, when the SPPs transmitted through a step formed by WS_2_ boundary, there would be energy loss. And the reason for the difference between “on-off” and “off-on” cases may come from the absorption by excitons of TMDC. For the “off-on” case, the energy was carried by SPPs imprinted by 633 nm, which is detuned from the excitons absorption transition. It is less lossy than the “on-off” case where the SPPs carried the signal of WS_2_ PL whose energy well matched with the absorption of WS_2_. Therefore, the “off-on” case seems more efficient than “on-off” case.


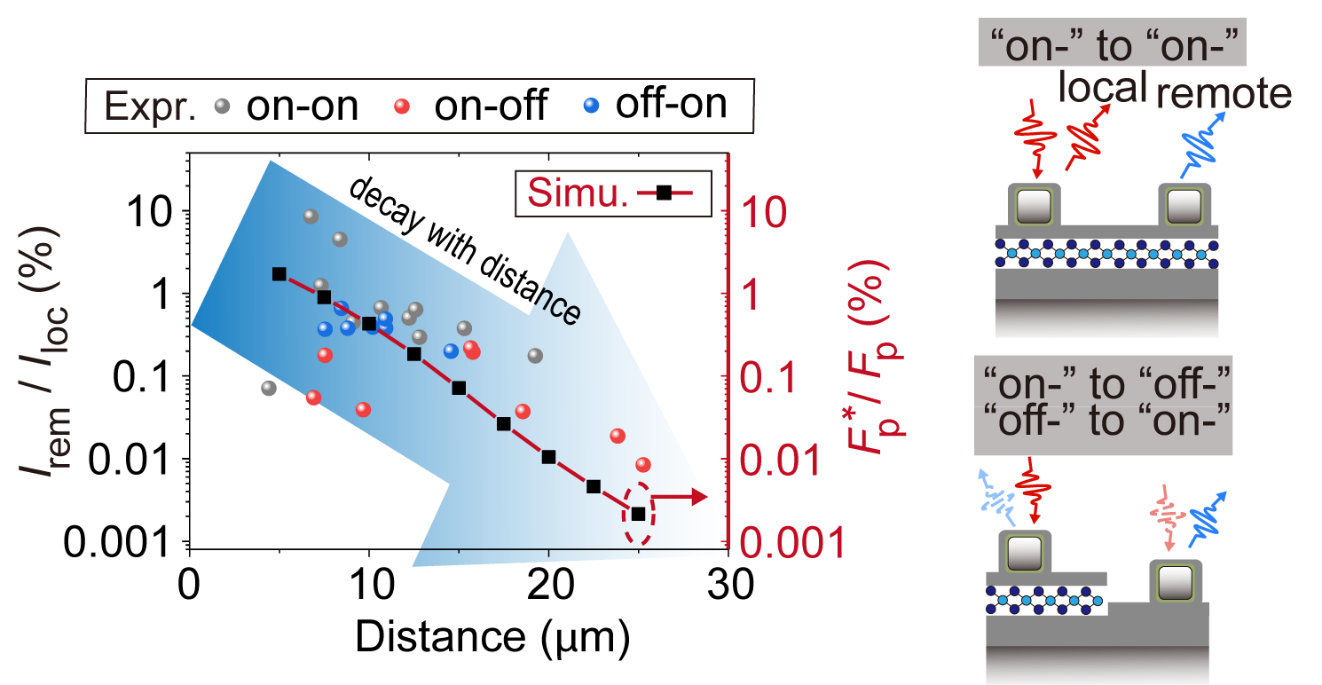


**Figure S4**: The efficiencies *β* = *I*_rem_/*I*_loc_ of the NCoM pairs we examined in Fig. S2. The data show a distinct decay manner with the increasing distance between NCoMs. The insets define the remote spectroscopy with different cases. Theoretical efficiencies by dividing “remote” Purcell factor *F*_p_* by the “local” Purcell factor *F*_p_ of NCoM pairs (*β*_cal_ = *F*_p_*/ *F*_p_) against various inter-particle distances match with the experimental results.

**S3.** **Angle dependence of the NCoMs**

The nanocubes were randomly drop-casted onto the substrate. Therefore, they would have random orientations. Here we calculated the dependence of the out-coupling efficiency (Fig. S5a) and remote Purcell factor *F*_p_* (Fig. S5b) from the differently-oriented NCoMs, which shows no angle dependence.

Figure S5a shows the *Q*_scat_ from the nanocube, whose definition is *Q*_scat_ = *P*_scat_/*P*_spp_. Boundary mode analysis in COMSOL was used to search the SPP mode on the substrate. This SPP was input from the port (see Fig. S5a inset) at the side boundary to mimic the experiment. *P*_spp_ is the energy that enters the physical area of the nanocube (~ 80 nm × 80 nm). And the *P*_scat_ is the energy scattered out from the NCoM. Figure S5b shows the remote Purcell factor (see Methods) from the transmitting NCoM with different orientations. Overall, the results show that there is no orientation dependence, which is robust.

**
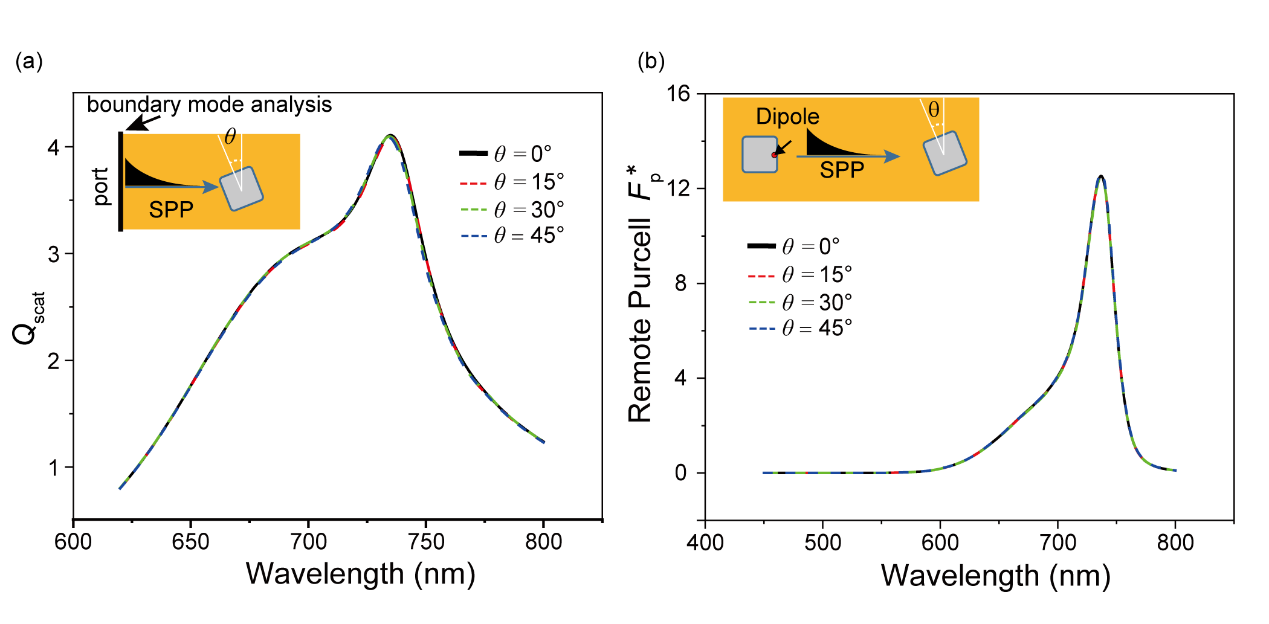
**

**Figure S5:** (a) Out-coupling efficiency from the NCoM with different orientations (*θ*) excited by SPPs at the port given by boundary mode analysis. (b) Remote Purcell factor *F*_p_* from the transmitting NCoM with different orientations, excited by putting a dipole under the receiving NCoM.

**S4. NCoM on the path hardly degrades the efficiency**

Here we want to stress that in many cases of Fig. S2, the energy transfer from the receiving to the transmission antenna seemed to be “blocked” by an extra nanocube (e.g., “a” to “b” in main text Fig. 3e). But we still observed prominent remote sensing performance. In Fig. S6, we calculated the case with and without the blocking nanocube, and the remote Purcell factor seemed to be only slightly decreased (~ 7%). We could also examine the electric field Fig. S6(b-e) that the extra nanocube won’t much alter the field (Fig. S6d and e).

**
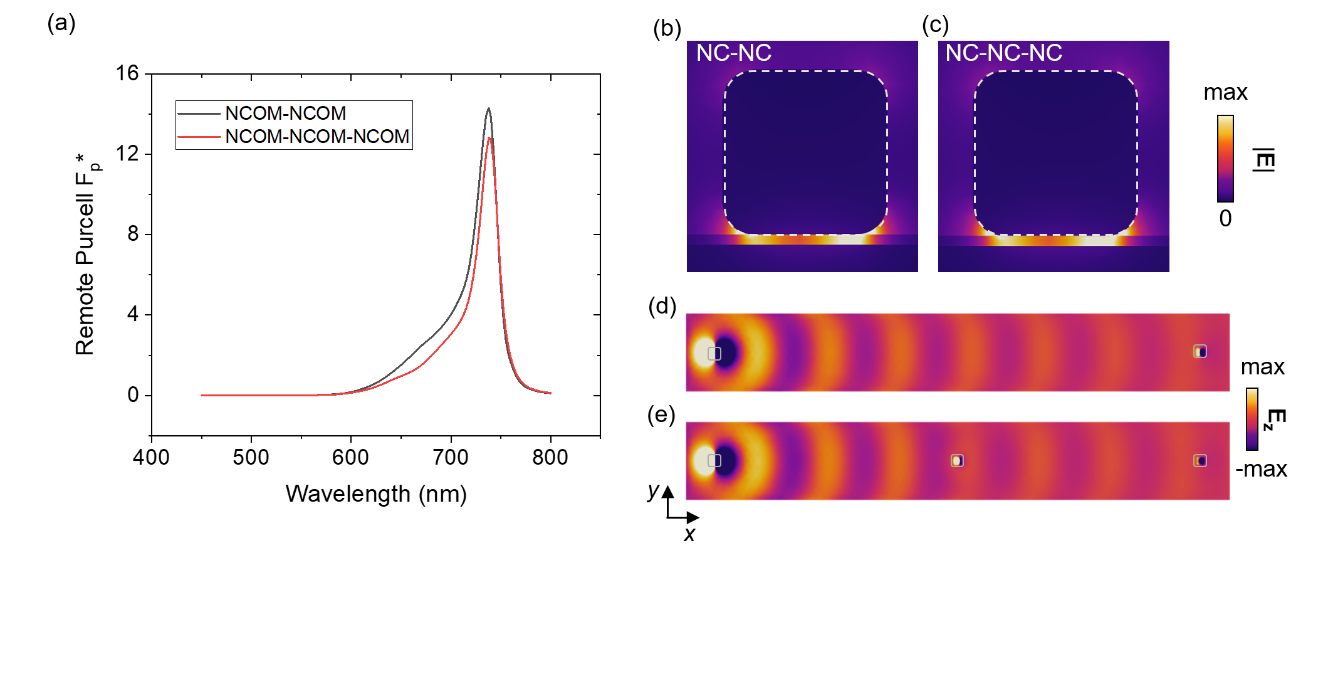
**

**Figure S6:** (a) Remote Purcell factors *F*_p_* from the transmission NCOM with (red) and without (black) an extra NCoM blocking in the pathway. (b, c) are the normalized electric field with (c) and without (b) a blocking nanocube. (d) and (e) are the *z*-electric field which presents the energy transfer via SPPs.

**S5. Comparison of** **NWoM pair with NCoM and NPoM pair**

**
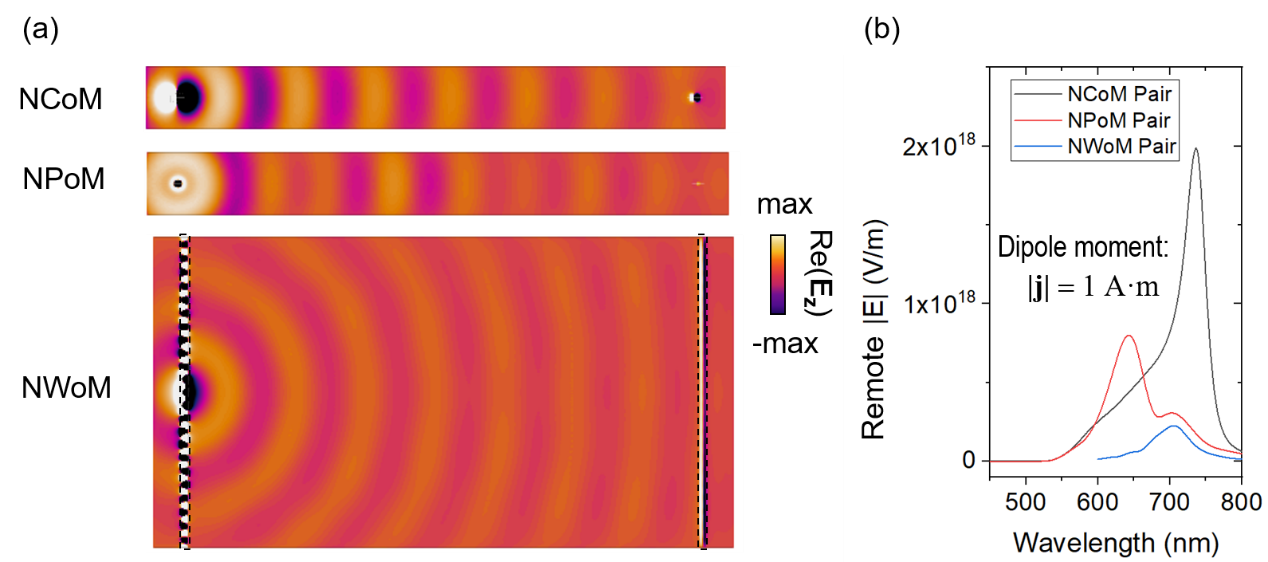
**

**Figure S7:** Comparison of the remote performances of the NWoM, NCoM and NPoM pairs: (a) electric field **E**_z_ distribution, (b) normalized electric field |**E|** under the remote antenna. Dipoles were inserted under the left receiving antennas. Three figures share the same colorbar. The NCoM has the largest electric field.

When comparing the remote performance of NCoM, NPoM and NWoM pairs, we could put a dipole (dipole moment 1 A·m) at the maximum-field-position under the receiving nanoantenna (left ones in Fig. S7a), then measure the electric field on the surface and under the transmitting antenna (right ones in Fig. S7a). These three configurations all have a separation of 5 μm. The fields (Fig. S7a) on the metal surface indicate the efficiency of the SPPs converted by the local near-field sources. Due to the same colorbar applied, we could intuitively find that NCoM pairs have the best in-coupling efficiency. NWoM has the least efficiency. Specifically, we could extract the electric field at the remote antenna excited by the *z*-direction dipole with the same dipole moment (Fig. S7b). The NCoM has the largest electric field while the NWoM has the least electric field. Therefore, NCoM pairs have the best remote performance in this form.

**S6. Size dependence of the NCoM pair’s remote Purcell factor**


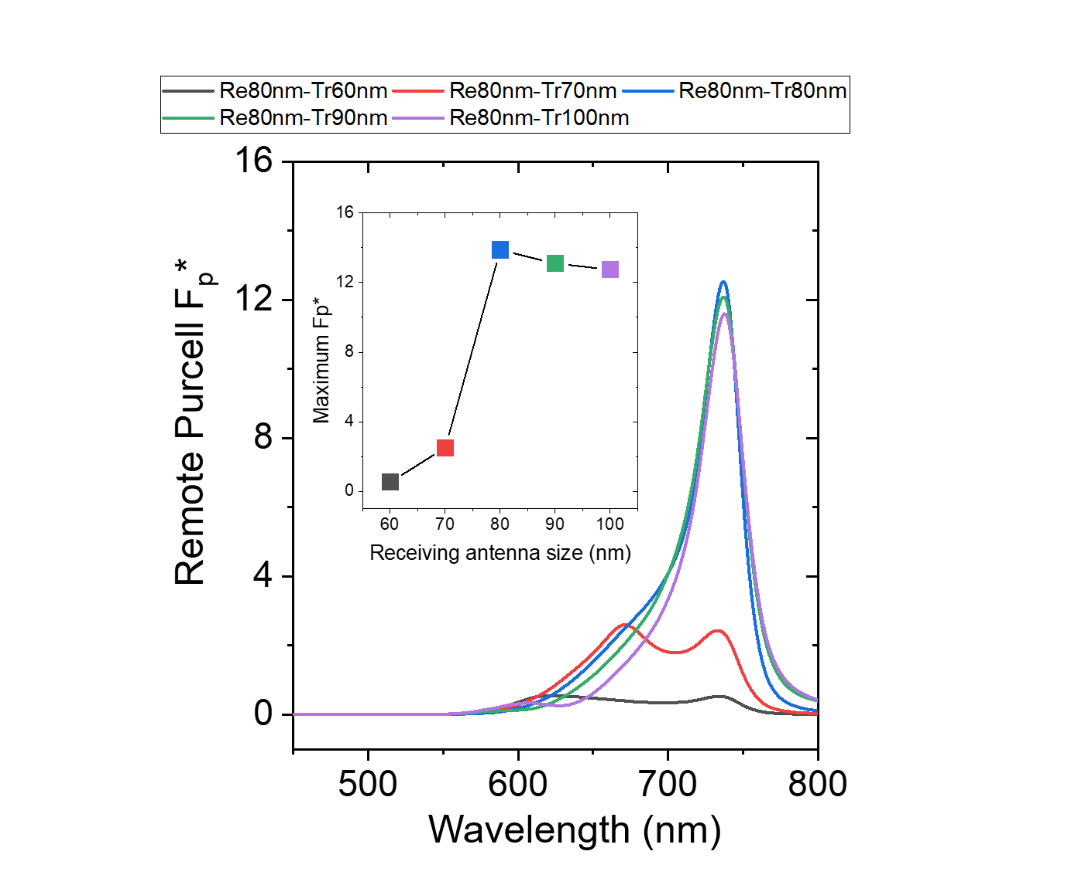


**Figure S8**: Remote Purcell factors *F*_p_* of NCoM pairs with 80 nm receiving antenna accomplished by transmitting antenna with different sizes (from 60 nm to 100 nm). Inset gives the maximum *F*_p_* of such pairs, showing an optimum efficiency when the receiving and transmitting antennas have identical sizes (80 nm here).

Size dependence is another significant issue in NCoM pair setup. Because the system relies on energy transfer via SPPs, it is intuitive that the receiving and transmitting antennas would have the best performance when they are on resonance. To prove that, we set the size of the receiving NCoM antenna as 80 nm and vary the size of the transmitting NCoM antenna from 60 nm to 100 nm (Fig. S8). The remote Purcell factor shows an increasing and decreasing trend against the size of the transmitting antenna. Matched sizes (receiving and transmitting NCoM having the same size ~ 80 nm) give the best *F*_p_*.
